# Enantioselective Synthesis of 2,2-Disubstituted Terminal Epoxides via Catalytic Asymmetric Corey-Chaykovsky Epoxidation of Ketones

**DOI:** 10.3390/molecules17021617

**Published:** 2012-02-07

**Authors:** Toshihiko Sone, Akitake Yamaguchi, Shigeki Matsunaga, Masakatsu Shibasaki

**Affiliations:** 1 Graduate School of Pharmaceutical Sciences, The University of Tokyo, 7-3-1 Hongo, Bunkyo-ku, Tokyo 113-0033, Japan; Email: toshihiko-sone@ds-pharma.co.jp (T.S.); yamaguchi.akitake.pz@daiichisankyo.co.jp (A.Y.); 2 Institute of Microbial Chemistry, Tokyo, Kamiosaki 3-14-23, Shinagawa-ku, Tokyo 141-0021, Japan

**Keywords:** asymmetric catalysis, asymmetric synthesis, epoxide, rare earth metal, sulfur ylide

## Abstract

Catalytic asymmetric Corey-Chaykovsky epoxidation of various ketones with dimethyloxosulfonium methylide using a heterobimetallic La-Li_3_-BINOL complex (LLB) is described. The reaction proceeded smoothly at room temperature in the presence of achiral phosphine oxide additives, and 2,2-disubstituted terminal epoxides were obtained in high enantioselectivity (97%–91% *ee*) and yield (>99%–88%) from a broad range of methyl ketones with 1-5 mol% catalyst loading. Enantioselectivity was strongly dependent on the steric hindrance, and other ketones, such as ethyl ketones and propyl ketones resulted in slightly lower enantioselectivity (88%–67% *ee*).

## 1. Introduction

**Scheme 1:**
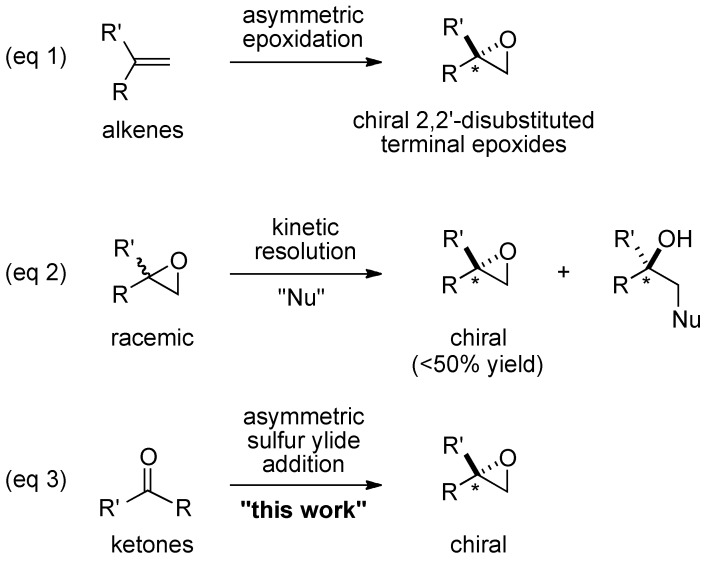
Approaches to chiral 2,2′-disubstituted terminal epoxides.

Optically active epoxides are versatile chiral building blocks for the efficient synthesis of natural products and biologically active compounds such as pharmaceuticals. Tremendous efforts have been devoted for the enantioselective synthesis of epoxides, and various useful catalytic asymmetric epoxidation methods have been reported [1,2]. 2,2-Disubstituted terminal epoxides, however, still remain particularly challenging target compounds. Catalytic asymmetric epoxidations of geminally disubstituted terminal unfunctionalized alkenes (eq 1, Scheme 1) have been studied using enzymes [3,4], chiral metal- and organo-catalysts [5,6,7], but the enantioselectivity, yield, and/or substrate generality of these reactions are not satisfactory. One of the best chiral catalysts for asymmetric epoxidation of geminally disubstituted terminal unfunctionalized alkenes was developed in 2008 by Shi and co-workers. In their system, good enantioselectivity was achieved with α-*t*-Bu-substituted styrene (86% *ee*), but smaller substituents, especially α-Me-substituted styrene, resulted in moderate enantioselectivity [8].

An elegant approach toward 2-monosubstituted terminal epoxides has also been accomplished via hydrolytic kinetic resolution with the Jacobsen’s salen-Co-complex [9], but the method has not been successfully applied to 2,2-disubstituted terminal epoxides. Instead, Jacobsen and coworkers reported a seminal work on the Cr-salen catalyzed kinetic resolution of 2,2-disubstituted terminal epoxides with azide nucleophile (eq 2, Scheme 1), but the substrate scope was limited to 2,2-dialkyl-substituted terminal epoxides [10,11,12].

**Figure 1:**
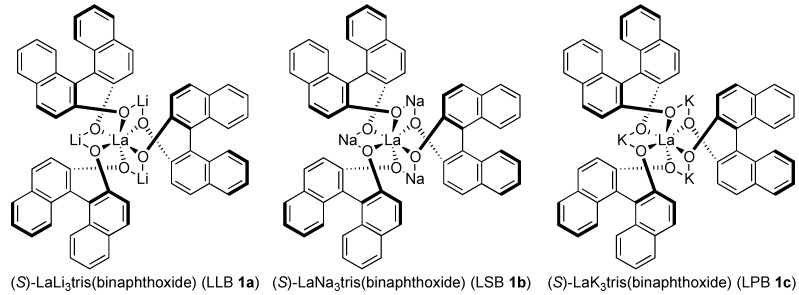
Structures of (*S*)-RE-M_3_-tris(binaphthoxide) complex (REMB, RE = rare earth), LLB **1a**, LSB **1b**, and LPB **1c**.

Considering the importance of 2,2-disubstituted epoxides as key building blocks for synthesizing valuable chiral tertiary alcohols, a new strategy to synthesize chiral 2,2-disubstituted terminal epoxides is highly desirable [13]. To address this issue, we previously communicated an alternative approach based on catalytic asymmetric Corey-Chaykovsky epoxidation [14] of ketones (eq 3, Scheme 1), *i.e.*, the addition of sulfur ylide to methyl ketones. A heterobimetallic La-Li_3_-tris(binaphthoxide) (LLB **1a**, Figure 1) complex worked nicely as a double Lewis acid two-center asymmetric catalyst, and provided 2,2-disubstituted terminal epoxides in high enantioselectivity from methyl ketones [15,16]. In this article, we report the full details of our work on this reaction.

## 2. Results and Discussion

### 2.1. Optimization Studies

**Figure 2:**
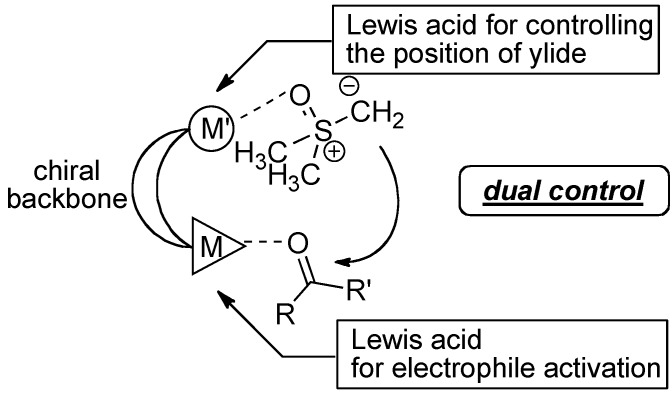
Working hypothesis to realize high enantio induction with doubly Lewis acidic chiral bimetallic/multimetallic catalysts.

For the synthesis of 2,2-disubstituted terminal epoxides via 1,2-addition of a sulfur ylide to ketones, dimethyloxosulfonium methylide (**2**) and acetophenone (**3a**) were selected as model substrates. In general, enantioselective addition of nucleophiles to simple non-activated ketones, like acetophenone (**3a**), is much more difficult than that to aldehydes. Indeed, when we started this project, there was only one report on catalytic asymmetric Corey-Chaykovsky epoxidation of ketones, in which ketone **3a** gave epoxide **4a** in only 23% *ee* [17]. Because steric difference of the substituents at the prochiral carbonyl group of ketones is much less than that of aldehydes, a chiral catalyst should have high enantio-differentiation ability to achieve high enantioselectivity. To achieve high stereocontrol, we hypothesized that the dual control of two reactants [18], ketones and sulfur ylide, by a doubly Lewis acidic bimetallic/multimetallic complexes would be beneficial over a conventional method based on the activation of ketone alone with a mono-metallic chiral Lewis acid catalyst. The working model using the double Lewis acid catalysts is shown in Figure 2.

On the basis of the working hypothesis in Figure 2, several chiral multimetallic catalysts developed in our group [19,20,21] were screened, and the initial screening revealed that heterobimetallic rare earth-alkali metal RE-M_3_-tris(binaphthoxide) complexes (REMB, Figure 1) [22,23,24,25] were the most promising candidates for the addition of a sulfur ylide to ketones. Optimization studies using REMB complexes are summarized in Table 1.

**Table 1:** Optimization of reaction conditions. 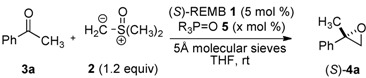

Entry	REMB catalyst	R: (mol%)	5	time (h)	% yield ^a^	% *ee*
1 ^b^	LLB **1a**	none		48	79	15
2	LLB **1a**	none		12	80	72
3	LSB **1b**	none		12	25	14
4	LPB **1c**	none		12	17	52
5	LLB **1a**	Ph- (5)	**5a**	12	77	80
6	LLB **1a**	4-Cl-C_6_H_4_- (5)	**5b**	12	99	74
7	LLB **1a**	C_6_F_5_- (5)	**5c**	12	61	48
8	LLB **1a**	*n-*butyl (5)	**5d**	12	87	75
9	LLB **1a**	cyclohexyl (5)	**5e**	12	99	76
10	LLB **1a**	2,4,6-Me_3_-C_6_H_2_- (5)	**5f**	12	97	75
11	LLB **1a**	4-MeO-C_6_H_4_- (5)	**5g**	12	82	77
12	LLB **1a**	2,6-(MeO)_2_-C_6_H_3_- (5)	**5h**	12	84	93
13	LLB **1a**	2,4,6-(MeO)_3_-C_6_H_2_- (5)	**5i**	12	98 ^c^	96
14	LLB **1a**	2,4,6-(MeO)_3_-C_6_H_2_- (10)	**5i**	12	94	95
15	LLB **1a**	2,4,6-(MeO)_3_-C_6_H_2_- (15)	**5i**	12	92	92

^a^ Yield determined by ^1^H-NMR analysis of crude mixture; ^b^ Reaction was run in the absence of MS 5Å. (*R*)-**4a** was obtained in major; ^c^ Isolated yield after purification by column chromatography.

LLB **1a** promoted the reaction at room temperature in 79% yield, but the enantioselectivity was poor (entry 1, 15% *ee*). In the presence of MS 5Å, enantioselectivity improved to 72% *ee* (entry 2). Other metal combinations, such as La-Na (LSB, **1b**) and La-K (LPB, **1c**), resulted in much less satisfactory yield and enantioselectivity (entry 3, 25% yield, 14% *ee*, entry 4, 17% yield, 52% *ee*). Several chiral biphenyldiol ligands, which were useful in a related catalytic asymmetric Corey-Chaykovsky cyclopropanation of enones with a heterobimetallic REMB-type complex [26], were screened, but resulted in lower enantioselectivity. Although a mixed alkali metal La-Li_2_-Na-(biphenyldiol)_3_ system gave the best enantioselectivity in the catalytic asymmetric Corey-Chaykovsky cyclopropanation, the mixed alkali metal system did not afford positive effects in the present epoxidation of ketones. Many trials to improve the enantioselectivity revealed that the addition of achiral phosphine oxide **5** was effective. In the presence of 5 mol% of Ph_3_P=O **5a**, **4a** was obtained in 80% *ee* (entry 5). Because steric and electronic modification of achiral phosphine oxides often had beneficial effects in other rare earth metal-catalyzed asymmetric reactions [27,28,29,30,31], various types of phosphine oxides were screened (entries 5–13). Sterically hindered and electron-rich aryl phosphine oxide, Ar_3_P=O **5i** (Ar = 2,4,6-trimethoxyphenyl) in entry 13, gave the best results, affording **4a** in 98% isolated yield and 96% *ee* after 12 h. A molar ratio of LLB **1a**: Ar_3_P=O **5i** was investigated in entries 13–15, and a 1:1 ratio was sufficient to achieve high enantioselectivity.

### 2.2. Substrate Scope and Limitations

The optimized reaction conditions using an LLB **1a**:Ar_3_P=O **5i** = 1:1 mixture were applicable to various ketones, as summarized in Table 2 (methyl ketones) and Table 3 (other ketones).

**Table 2:** Catalytic asymmetric synthesis of 2,2-disubstituted terminal epoxides from various methyl ketones ^a^. 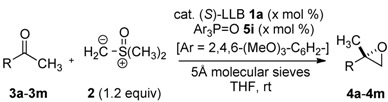

Entry	Ketone: R	3	Epoxide 4	Cat. (x mol%)	Time (h)	% Yield ^b^	% *ee*
1	Ph	**3a**	**4a**	5	12	98	96
2	2-naphthyl	**3b**	**4b**	5	12	97	96
3	2-naphthyl	**3b**	**4b**	2.5	18	96	94
4	2-naphthyl	**3b**	**4b**	1	60	96	92
5	4-Cl-C_6_H_4_	**3c**	**4c**	5	12	>99	94
6	3-Cl-C_6_H_4_	**3d**	**4d**	5	12	>99	94
7	2-Cl-C_6_H_4_	**3e**	**4e**	5	12	96	95
8	4-F-C_6_H_4_	**3f**	**4f**	5	12	94	97
9	4-EtO_2_C-C_6_H_4_	**3g**	**4g**	5	12	94	94
10^c^	4-Me-C_6_H_4_	**3h**	**4h**	5	12	97	92
11	3-pyridyl	**3i**	**4i**	5	12	97	92
12	PhCH_2_CH_2_-	**3j**	**4j**	5	12	99 ^e^	93
13	*n*-octyl	**3k**	**4k**	5	12	>99	93
14 ^c^	cyclohexyl	**3l**	**4l**	5	12	88 ^d^	96
15	4-EtO_2_C-(CH_2_)_3_-	**3m**	**4m**	5	12	>99	91

^a^ Reaction was performed in THF (0.1 M on ketone **3**) at room temperature (20–23 °C) with MS 5Å. 1.2 equiv. of ylide **2** prepared from trimethyloxosulfonium chloride and NaH were used; ^b^ Isolated yield after purification by column chromatography; ^c^ Enantiomeric excess was determined after epoxide ring opening, see Experimetal Section for detail; ^d^ NMR yield was >95%, but the isolated yield decreased because epoxide **4l** was volatile.

Aryl methyl ketones **3a**–**h** gave epoxides in >99%–94% yield and 97%–92% *ee* (Table 2, entries 1–10). Although long reaction times were required, catalyst loading was successfully reduced to 2.5 mol% and 1 mol%, while retaining good enantioselectivity (entries 3 and 4). Acetophenone derivatives **3c**–**e** with an electron-withdrawing substituent at either the *para*-, *meta*- or *ortho*-positions gave epoxides in high yield and enantioselectivity (entries 5–7).

**Table 3:** Catalytic asymmetric synthesis of 2,2-disubstituted terminal epoxides from other ketones ^a^. 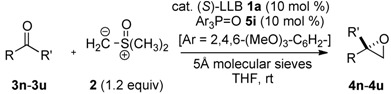

Entry	Ketone: R	R′	3	Epoxide 4	Time (h)	% Yield ^b^	% *ee*
1	Ph	Et	**3n**	**4n**	18	91	88
2	4-Cl-C_6_H_4_	Et	**3o**	**4o**	18	94	87
3	4-Br-C_6_H_4_	Et	**3p**	**4p**	18	92	85
4	3-Cl-C_6_H_4_	Et	**3q**	**4q**	18	96	81
5	2-F-C_6_H_4_	Et	**3r**	**4r**	18	88	67
6	3-pyridyl	Et	**3s**	**4s**	18	89	73
7	4-Cl-C_6_H_4_	*n*Pr	**3t**	**4t**	18	90	73
8	Ph	*i*Pr	**3u**	**4u**	36	60	70

^a^ Reaction was performed in THF (0.1 M on ketone **3**) at room temperature (20–23 °C) with MS 5Å. 1.2 equiv. of ylide **2** prepared from trimethyloxosulfonium chloride and NaH were used; ^b^ Isolated yield after purification by column chromatography.

It is noteworthy that high yield and high enantioselectivity were achieved, even with *ortho*-substituted ketone **3e**. The broad generality of aryl methyl ketones is particularly useful from synthetic point of view, because the methods for producing chiral 2-aryl-2-methyl terminal epoxides in high enantioselectivity have heretofore been limited to biocatalytic kinetic resolution approaches [11,12]. It is also noteworthy that ketones bearing a Lewis basic moiety, *i.e.*, ketone **3g** with an ester functional group and pyridyl methyl ketone **3i**, were applicable even under the Lewis acid-stereocontrol conditions, and epoxides **4g** and **4i** were obtained in 94% *ee* and 92% *ee*, respectively (entries 9 and 11). The present catalyst also gave high enantioselectivity with alkyl methyl ketones. Not only α-branched alkyl ketone **3l**, but also linear alkyl ketones **3j** and **3k**, in which steric difference of two substituents at the prochiral carbonyl group is small, gave products in high enantioselectivity (93%–96% *ee*, entries 12–14).

In contrast to the methyl ketones listed in Table 2, other aryl alkyl ketones, such as propiophenone (**3n**), resulted in much lower reactivity and enantioselectivity. As summarized in Table 3, 10 mol% of catalyst was utilized to obtain products in synthetically useful yields. Ketones with a substituent at either the *para*- or *meta-*position gave product in 81%–87% *ee* (Table 3, entries 2–4). On the other hand, *ortho*-substituted ketone **3r** and pyridyl ketone **3s** resulted in lower enantioselectivity, 67% *ee* and 73% *ee*, respectively (entries 5–6). The reactivity of *i*-propyl ketone **3u** was much lower than other ketones possibly due to steric hindrance, and product **4u** was obtained in only 60% yield even after prolonged reaction time (entry 8, 36 h). The results in Table 2 and Table 3 indicate that the present method is complementary to Shi’s approach via catalytic asymmetric epoxidation of alkenes [8], in which alkenes with a bulkier substituent, such as *t*-Bu group, gave better enantioselectivity in comparison to those with smaller groups like Me and Et.

### 2.3. Postulated Role of Phosphine Oxide Additive

**Figure 3:**
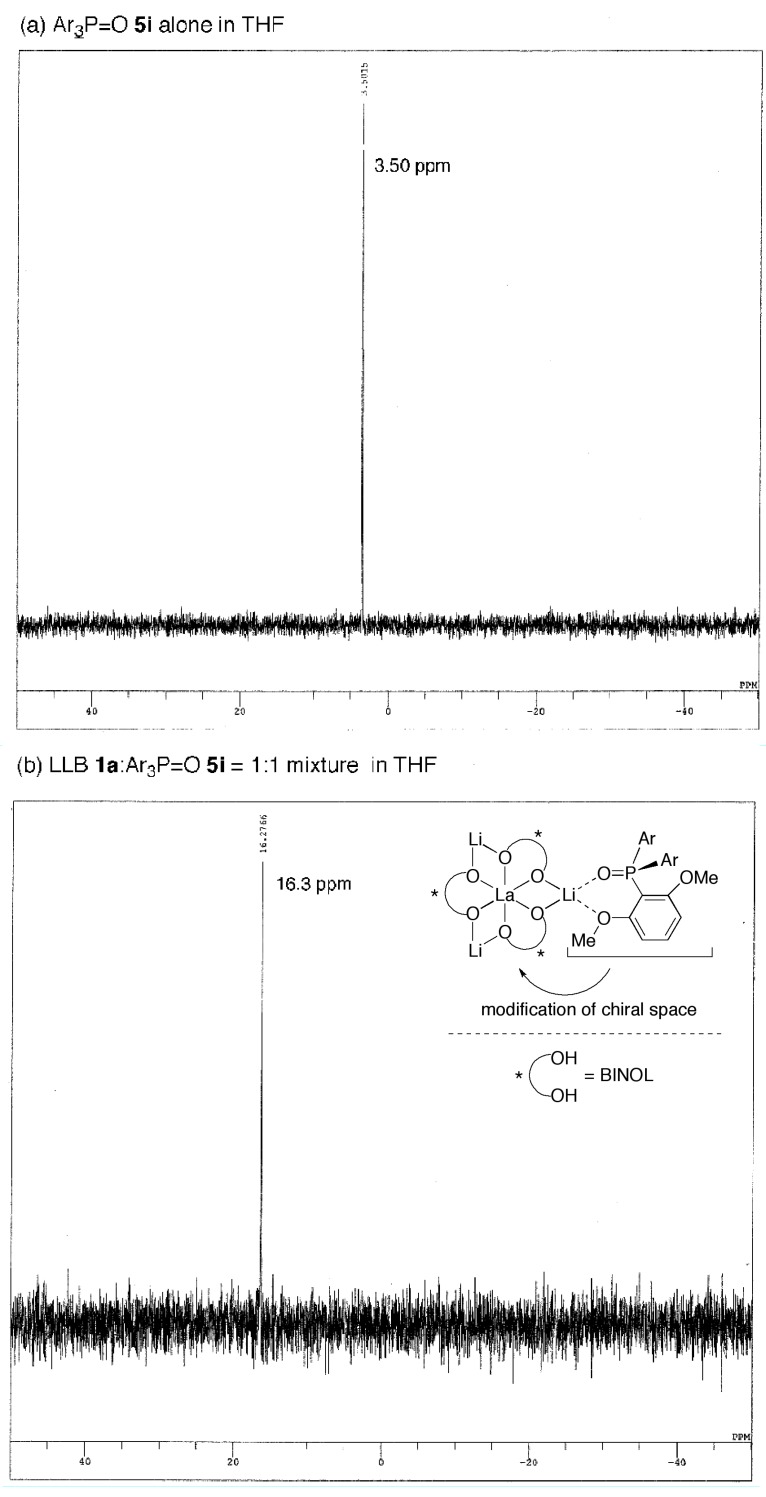
^31^P-NMR of (a) Ar_3_P=O **5i** alone and (b) Ar_3_P=O **5i** with LLB (16.3 ppm).

In the present system, the best yield and enantioselectivity were obtained with Ar_3_P=O **5i** additive. The results shown in Table 1, entries 5–13, suggested that the electron-donating and coordinating MeO-substituents at the 2,6-positions were key to improve enantioselectivity. ^31^P-NMR analysis of Ar_3_P=O **5i** alone (3.50 ppm) and Ar_3_P=O **5i** with LLB (16.3 ppm) indicated that Ar_3_P=O **5i** coordinates to LLB **1a** to form the LLB:Ar_3_P=O **5i** = 1:1 complex, which would be the active species in the present system (Figure 3). On the basis of several previous reports on steric and electronic modification of REMB catalysts with achiral phosphine oxides [29], we believe that electron-rich, bulky, and chelating achiral additive **5i** suitably modified the chiral environment of LLB [32,33,34,35], resulting in better yield and enantioselectivity.

### 2.4. Transformation of Epoxide

**Scheme 2:**
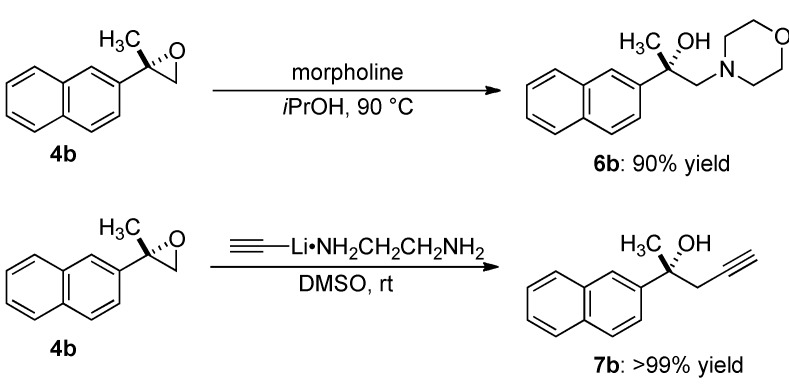
Regioselective ring-opening of 2,2-disubstituted terminal epoxide.

To demonstrate the synthetic utility of epoxides, transformations of products into chiral tertiary-alcohols were investigated (Scheme 2). Ring-opening of epoxide with amine nucleophile proceeded selectively at the terminal position in isopropanol at 90 °C, giving β-amino *tert*-alcohol **6b** in 90% yield. Reaction with alkynyl lithium reagent also proceeded regioselectively, and afforded **7b** in >99% yield.

## 3. Experimental

### 3.1. General

Infrared (IR) spectra were recorded on a JASCO FT/IR 410 Fourier transform infrared spectrophotometer. NMR spectra were recorded on a JEOL JNM-LA500 spectrometer, operating at 500 MHz for ^1^H-NMR and 125.65 MHz for ^13^C-NMR. Chemical shifts in CDCl_3_ were reported downfield from TMS (=0 ppm) for ^1^H-NMR. For ^13^C-NMR, chemical shifts were reported downfield from TMS (=0 ppm) or in the scale relative to CHCl_3_ (77.00 ppm for ^13^C-NMR) as an internal reference. Optical rotations were measured on a JASCO P-1010 polarimeter. ESI mass spectra were measured on Waters micromass ZQ. FAB mass spectra (for HRMS) were measured on a JEOL JMS-700 spectrometer. The enantiomeric excess (*ee*) was determined by HPLC analysis or GC analysis. HPLC was performed on JASCO HPLC systems consisting of the following: pump, PU-2080; detector, UV-2075, measured at 210 nm, 220 nm, or 254 nm; column, DAICEL CHIRALCEL OJ-H, DAICEL CHIRALPAK AD-H or DAICEL CHIRALCEL OD; mobile phase, hexane–2-propanol; flow rate, 0.5 mL/min or 1.0 mL/min. GC analysis was performed Shimadzu GC-14A with Varian Chirasil DEX CB column (0.25 mm × 25 m). Reactions were carried out using flame-dried glasswares in dry solvents under an argon atmosphere, unless otherwise stated. La(O-*i*Pr)_3_ was purchased from Kojundo Chemical Laboratory Co., LTD., 5-1-28, Chiyoda, Sakado-shi, Saitama 350-0214, Japan. (O-*i*Pr)_3_ with the same quality is also available from Aldrich. MS 5Å (Molecular Sieve UOP 5A, powder) was purchased from Fluka. Trimethyloxosulfonium chloride was purchased from Aldrich and used as received. Bromide-free MeLi in hexane was purchased from Kanto Chemicals, and was titrated prior to use. Column chromatography was performed with silica gel 60N (40–100 μm spherical, neutral). Tetrahydrofuran (THF) was distilled from sodium benzophenone ketyl. Other reagents were purified by the usual methods.

### 3.2. Preparation of (*S*)-La-Li_3_-(binaphthoxide)_3_ (**1a**) Catalyst Solution

To a stirred solution of (*S*)-BINOL (1.288 g, 4.50 mmol) in THF (7.5 mL) at 0 °C was added slowly a solution of La(O-*i*Pr)_3_ (7.5 mL, 1.50 mmol, 0.20 M in THF). The ice-bath was removed and the solution was stirred for 1 h at room temperature. Then, THF and *i*-PrOH was removed under reduced pressure and dried for 8 h under vacuum at room temperature. The residue was cooled at 0 °C, and THF (7.5 mL) was added. To the solution was added slowly MeLi (4.13 mL, 4.50 mmol, 1.09 M in hexane, bromide-free quality, purchased from Kanto Chemical; a freshly opened bottle was used). The mixture was stirred at room temperature for 12 h, and then THF was removed under reduced pressure and dried for 8 h under vacuum at room temperature. The residue was cooled at 0 °C, and THF (7.5 mL) was added to afford (*S*)-La-Li_3_-(binaphthoxide)_3_ solution (0.1 M in THF). The catalyst solution (0.1 M in THF) was stored at room temperature under Ar atmosphere, and used for asymmetric reactions. The activity and selectivity of the catalyst solution remained unchanged at least for three months.

### 3.3. Preparation of Dimethyloxosulfonium Methylide (**2**) Solution

Sodium hydride (1.58 g) as a mineral dispersion was placed in a 100 mL, two-necked round-bottomed flask and washed three times with portions of dry petroleum ether (10 mL each) by swirling, allowing the hydride to settle, and decanting in order to remove the mineral oil. The flask was immediately fitted with reflux condenser and a glass stopper, and evacuated to remove the last traces of petroleum ether (1.20 g, 50.0 mmol of pure NaH was obtained). After refilling with Ar, trimethyloxosulfonium chloride (7.58 g, 58.9 mmol) and dry THF (50 mL) were added. With stirring, the mixture was heated to reflux with oil bath (bath temp: 80–90 °C). The evolution of hydrogen gas was fairly rapid at first, but after several minutes it ceased. After ca. 2 h, rapid hydrogen evolution again began and the reaction was finished as was evidenced by lack of hydrogen evolution. After refluxing for 3.0–5.5 h, a milky-white suspension was obtained. The mixture was cooled at 0 °C for 1.5 h, and filtered through a pad of dried Celite under Ar, directly into a flame dried storage flask to afford pale yellow clear solution (0.750 M, determined by titration).

### 3.4. General Procedure for Catalytic Asymmetric 1,4-Additions of β-Keto Esters to Nitroalkenes under Solvent-Free Conditions

5Å molecular sieves (150 mg) in a test tube were flame dried under reduced pressure (ca. 1 mmHg). After cooling down to room temperature, tris(2,4,6-trimethoxyphenyl)phosphine oxide (**5i**, 0.015 mmol, 8.23 mg), (*S*)-La-Li_3_-(binaphthoxide)_3_ (**1a**, 0.1 M in THF solution, 150 μL, 0.015 mmol) and THF (2.37 mL) were added at room temperature. After being stirred for 30 min at room temperature, dimethyloxosulfonium methylide (**2**, 0.36 mmol, 480 μL, 0.750 M in THF) was added to the mixture. After being stirred for 5 min at room temperature, ketone **3** (0.30 mmol) was added. After 12 h, NH_4_Cl *aq*. and Et_2_O were added. The water layer was extracted with Et_2_O (×2). The combined organic layers were washed with brine, and dried over Na_2_SO_4_. After evaporating solvent under reduced pressure, the residue was purified by flash column chromatography (neutral SiO_2_, hexane/diethyl ether) to give product **4** [**Caution:*** Most of the epoxides are somewhat unstable under acidic conditions. In order to avoid epoxide ring opening during purification, flash column chromatography was conducted with the injection part cooled by dry ice*].

#### 3.4.1. (2*S*)-2-Methyl-2-phenyloxirane (**4a**)

Colorless oil; IR (neat) ν 3034, 2984, 1446, 1060, 758, 698 cm^−1^; ^1^H-NMR (CDCl_3_) δ 7.38–7.32 (m, 4H), 7.29–7.27 (m, 1H), 2.98 (d, *J *= 5.3 Hz, 1H), 2.81 (d, *J* = 5.3 Hz, 1H) 1.73 (s, 3H); ^13^C-NMR (CDCl_3_) δ 141.2, 128.3, 127.4, 125.3, 57.0, 56.7, 21.8; ESI-MS *m*/*z* 157 [M+Na]^+^; HRMS [ESI] Calcd for C_9_H_11_O^+^ [M+H]^+^: 135.0810; Found 135.0807; The enantiomeric excess of **4a** was determined by chiral stationary-phase HPLC analysis [DAICEL CHIRALCEL OD, *i*-PrOH/hexane 1/99, flow rate 0.5 mL/min, *t*_R_ 11.5 min (minor, 2.2%) and 13.9 min (major, 97.8%), detection at 210 nm]; [α]_D_^24.3^ +20.4 (*c* 1.15, CHCl_3_). Absolute configuration of **4a** was determined to be (*S*) by comparing the sign of optical rotation with the literature data. Lit. [α]_D_^20^ −20.1 (*c* 1.02, CHCl_3_) for (*R*)-**4a** [36].

#### 3.4.2. (2*S*)-2-Methyl-2-naphthalen-2-yloxirane (**4b**)

Colorless solid; IR (KBr) ν 3051, 2978, 1384, 865, 826, 742 cm^−1^; ^1^H-NMR (CDCl_3_) δ 7.86 (d, *J* = 1.6 Hz, 1H), 7.84–7.81 (m, 3H), 7.50–7.44 (m, 3H), 3.06 (d, *J* = 5.5 Hz, 1H), 2.90 (d, *J* = 5.5 Hz, 1H), 1.83 (s, 3H); ^13^C-NMR (CDCl_3_) δ 138.6, 133.1, 132.7, 128.1, 127.9, 127.5, 126.2, 125.9, 124.4, 123.1, 57.0, 56.9, 21.8; ESI-MS *m*/*z* 207 [M+Na]^+^; HRMS [ESI] Calcd for C_13_H_13_O^+^ [M+H]^+^: 185.0966; Found 185.0959; The enantiomeric excess of **4b** was determined by chiral stationary-phase HPLC analysis [DAICEL CHIRALCEL OJ-H, *i*-PrOH/hexane 0.5/99.5, flow rate 0.5 mL/min, *t*_R_ 44.5 min (minor, 2.0%) and 48.8 min (major, 98.0%), detection at 254 nm]; [α]_D_^25.5^ +2.2 (*c* 1.00, CHCl_3_).

#### 3.4.3. (2*S*)-2-(4-Chlorophenyl)-2-methyloxirane (**4c**) [36]

Colorless oil; IR (neat) ν 3044, 2985, 1495, 1404, 1097, 827 cm^−1^; ^1^H-NMR (CDCl_3_) δ 7.29 (s, 4H), 2.97 (d, *J* = 5.4 Hz, 1H), 2.76 (d, *J* = 5.4 Hz, 1H), 1.69 (s, 3H); ^13^C-NMR (CDCl_3_) δ 139.8, 133.3, 128.5, 126.7, 57.0, 56.3, 21.6; ESI-MS *m*/*z* 191 [M+Na]^+^; HRMS [ESI] Calcd for C_9_H_10_ClO^+^ [M+H]^+^: 169.0420; Found 169.0417; The enantiomeric excess of **4c** was determined by GC analysis [Chirasil DEX CB, injector temp. = 200 °C, detector temp. = 250 °C, column temp. = 90 °C (isothermic), t = 32.4 min (minor, 2.9%), 35.3 min (major, 97.1%)]; [α]_D_^24.5^ +19.9 (*c* 1.25, CHCl_3_).

#### 3.4.4. (2*S*)-2-(3-chlorophenyl)-2-methyloxirane (**4d**)

Colorless oil; IR (neat) ν 2986, 2930, 1598, 1416, 1065, 784 cm^−1^; ^1^H-NMR (CDCl_3_) δ 7.37 (bs, 1H), 7.30–7.24 (m, 3H), 2.99 (d, *J* = 5.5 Hz, 1H), 2.78 (d, *J* = 5.5 Hz, 1H), 1.72 (s, 3H); ^13^C-NMR (CDCl_3_) δ 143.4, 134.4, 129.6, 127.6, 125.6, 123.5, 56.9, 56.3, 21.5; ESI-MS *m*/*z* 169 [M+H]^+^; The enantiomeric excess of **4d** was determined by GC analysis [Chirasil DEX CB, injector temp. = 200 °C, detector temp. = 250 °C, column temp. = 90 °C (isothermic), t = 24.1 min (minor, 3.1%), 25.0 min (major, 96.9%)]; [α]_D_^25.3^+10.5 (*c* 1.35, CHCl_3_).

#### 3.4.5. (2*S*)-2-(2-Chlorophenyl)-2-methyloxirane (**4e**)

Colorless oil; IR (neat) ν 3049, 2985, 1432, 1044, 865, 756 cm^−1^; ^1^H NMR (CDCl_3_) δ 7.49 (dd, *J* = 2.1, 7.0 Hz, 1H), 7.33 (dd, *J* = 1.6, 7.6 Hz, 1H), 7.27–7.21 (m, 2H), 3.01 (d, *J* = 5.2 Hz, 1H), 2.82 (d, *J* = 5.2 Hz, 1H), 1.66 (s, 3H); ^13^C-NMR (CDCl_3_) δ 139.6, 132.2, 129.1, 128.8, 128.4, 126.8, 57.8, 55.1, 22.7; ESI-MS *m*/*z* 191 [M+Na]^+^; HRMS [ESI] Calcd for C_9_H_10_OCl^+^ [M+H]^+^: 169.0420; Found 169.0423; The enantiomeric excess of **4e** was determined by chiral stationary-phase HPLC analysis [DAICEL CHIRALCEL OD, *i*-PrOH/hexane 1/99, flow rate 0.5 mL/min, *t*_R_ 9.07 min (minor, 2.6%) and 11.4 min (major, 97.4%), detection at 210 nm]; [α]_D_^27.2^ +95.0 (*c* 1.23, CHCl_3_).

#### 3.4.6. (2*S*)-2-(4-Fluorophenyl)-2-methyloxirane (**4f**) [36]

Colorless oil; IR (neat) ν 3047, 2986, 1606, 1513, 1223, 836 cm^−1^; ^1^H-NMR (CDCl_3_) δ 7.32 (dd, *J* = 5.2, 8.9 Hz, 2H), 7.01 (d, *J* = 8.9 Hz, 2H), 2.97 (d, *J* = 5.4 Hz, 1H), 2.77 (d, *J* = 5.4 Hz, 1H), 1.70 (s, 3H); ^13^C-NMR (CDCl_3_) δ 163.1, 161.2, 136.9, 136.9, 127.1, 127.0, 115.2, 1115.1, 56.9, 56.4, 21.9; ESI-MS *m*/*z* 175 [M+Na]^+^; HRMS [ESI] Calcd for C_9_H_10_FO^+ ^[M+H]^+^: 153.0716; Found 153.0711; The enantiomeric excess of **4f** was determined by GC analysis [Chirasil DEX CB, injector temp. = 200 °C, detector temp. = 250 °C, column temp. = 70 °C (isothermic), t = 24.1 min (minor, 1.7%), 26.8 min (major, 98.3%)]; [α]_D_^25.5^ +14.2 (*c* 1.35, CHCl_3_). 

#### 3.4.7. Ethyl 4-[(2*S*)-2-methyloxiran-2-yl]benzoate (**4g**)

Colorless oil; IR (neat) ν 2983, 1716, 1274, 1106, 1020, 771 cm^−1^; ^1^H-NMR (CDCl_3_) δ 8.01 (d, *J* = 8.9 Hz, 2H), 7.43 (d, *J* = 8.9 Hz, 2H), 4.37 (q, *J* = 7.4 Hz, 2H), 3.00 (d, *J* = 5.4 Hz, 1H), 2.78 (d, *J* = 5.4 Hz, 1H), 1.73 (s, 3H), 1.39 (t, *J* = 7.4 Hz, 3H); ^13^C-NMR (CDCl_3_) δ 166.3, 146.2, 129.6, 129.6, 125.2, 60.9, 57.1, 56.5, 21.4, 14.3; ESI-MS *m*/*z* 229 [M+Na]^+^; HRMS [ESI] Calcd for C_12_H_15_O_3_^+ ^[M+H]^+^: 207.1021; Found 207.1012; The enantiomeric excess of **4g** was determined by chiral stationary-phase HPLC analysis [DAICEL CHIRALCEL OD, *i*-PrOH/hexane 2/98, flow rate 0.5 mL/min, *t*_R_ 13.1 min (major, 97.2%) and 22.4 min (minor, 2.8%), detection at 254 nm]; [α]_D_^25.1^ +24.8 (*c* 1.55, CHCl_3_).

#### 3.4.8. (2*S*)-2-Methyl-2-(4-methylphenyl)oxirane (**4h**)

Colorless oil; IR (neat) ν 2982, 2924, 1517, 1066, 864, 816 cm^−1^; ^1^H-NMR (CDCl_3_) δ 7.25 (d, *J* = 8.1 Hz, 2H), 7.15 (d, *J* = 8.1 Hz, 2H), 2.96 (d, *J* = 5.4 Hz, 1H), 2.80 (d, *J* = 5.4 Hz, 1H), 2.34 (s, 3H), 1.70 (s, 3H); ^13^C-NMR (CDCl_3_) δ 138.1, 137.1, 129.0, 125.2, 57.0, 56.7, 21.8, 21.0; ESI-MS *m*/*z* 171 [M+Na]^+^; HRMS [ESI] Calcd for C_10_H_13_O^+ ^[M+H]^+^: 149.0966; Found 149.0963; [α]_D_^28.3^ +18.4 (*c* 1.17, CHCl_3_); The enantiomeric excess of **4h** was determined after the conversion into morpholinoalcohol by the similar procedure for **6b** synthesis. Chiral stationary-phase HPLC analysis: [DAICEL CHIRALPAK AD-H, *i*-PrOH/hexane 1/9, flow rate 1.0 mL/min, *t*_R_ 5.7 min (minor, 3.8%) and 8.4 min (major, 96.2%), detection at 210 nm].

#### 3.4.9. 3-[(2*S*)-2-Methyloxiran-2-yl]pyridine (**4i**)

Colorless oil; IR (neat) ν 3405, 2985, 1419, 1024, 809, 713 cm^−1^; ^1^H-NMR (CDCl_3_) δ 8.64 (dd, *J* = 0.7, 2.3 Hz, 1H), 8.52 (dd, *J* = 1.7, 4.9 Hz, 1H), 7.64 (ddd, *J* = 1.7, 2.3, 8.0 Hz, 1H), 7.25 (ddd, *J* = 0.7, 4.9, 8.0 Hz, 1H), 3.00 (d, *J* = 5.3 Hz, 1H), 2.80 (d, *J* = 5.3 Hz, 1H), 1.73 (s, 3H).; ^13^C-NMR (CDCl_3_) δ 148.8, 147.3, 136.7, 132.8, 123.1, 56.8, 55.2, 21.4; ESI-MS *m*/*z* 136 [M+H]^+^; HRMS [ESI] Calcd for C_8_H_10_NO^+^ [M+H]^+^: 136.0762; Found 136.0756; The enantiomeric excess of **4i** was determined by chiral stationary-phase HPLC analysis [DAICEL CHIRALCEL OD, *i*-PrOH/hexane 2/98, flow rate 0.5 mL/min, *t*_R_ 37.2 min (minor, 3.8%) and 58.0 min (major, 96.2%), detection at 254 nm]; [α]_D_^27.4^ +7.1 (*c* 1.07, CHCl_3_).

#### 3.4.10. (2*S*)-2-methyl-2-(2-phenylethyl)oxirane (**4j**) [10]

Colorless oil; IR (neat) ν 3027, 2928, 1454, 1064, 750, 700 cm^−1^; ^1^H-NMR (CDCl_3_) δ 7.38–7.34 (m, 2H), 7.28–7.26 (m, 3H), 2.82-2.78 (m, 2H), 2.69 (d, *J* = 4.9 Hz, 1H), 2.66 (d, *J* = 4.9 Hz, 1H), 2.01 (ddd, *J* = 6.4, 9.8, 16.4 Hz, 1H), 1.91 (ddd, *J* = 7.3, 9.5, 16.4 Hz, 1H), 1.46 (s, 3H); ^13^C-NMR (CDCl_3_) δ 141.6, 128.4, 128.2, 125.9, 56.7, 53.9, 38.5, 31.4, 21.0; ESI-MS *m*/*z* 185 [M+Na]^+^; The enantiomeric excess of **4j** was determined by chiral stationary-phase HPLC analysis [DAICEL CHIRALCEL OD, *i*-PrOH/hexane 2/98, flow rate 0.5 mL/min, *t*_R_ 13.1 min (minor, 3.4%) and 17.3 min (major, 96.6%), detection at 210 nm]; [α]_D_^24.5^ +2.9 (*c* 1.13, CHCl_3_). 

#### 3.4.11. (2*S*)-2-Methyl-2-octyloxirane (**4k**) [37]

Colorless oil; IR (neat) ν 2927, 2856, 1457, 1388, 1071, 798 cm^−1^; ^1^H-NMR (CDCl_3_) δ 2.60 (d, *J* = 4.9 Hz, 1H), 2.57 (d, *J* = 4.9 Hz, 1H), 1.61–1.55 (m, 1H), 1.50–1.42 (m, 1H), 1.40–1.24 (m, 12H), 1.30 (s, 3H), 0.88 (t, *J* = 7.4 Hz, 3H); ^13^C-NMR (CDCl_3_) δ 57.1, 53.9, 36.7, 31.8, 29.7, 29.5, 29.2, 25.2, 22.6, 20.9, 14.1; ESI-MS *m*/*z* 193 [M+Na]^+^; The enantiomeric excess of **4k** was determined by GC analysis [Chirasil DEX CB, injector temp. = 200 °C, detector temp. = 250 °C, column temp. = 70 °C (isothermic), t = 55.5 min (minor, 3.3%), 57.0 min (major, 96.7%)]; [α]_D_^24.6^ +5.9 (*c* 1.15, CHCl_3_).

#### 3.4.12. (2*S*)-2-Cyclohexyl-2-methyloxirane (**4l**) [10]

Colorless oil; IR (neat) ν 2927, 2854, 1449, 821 cm^−1^; ^1^H-NMR (CDCl_3_) δ 2.60 (d, *J* = 4.9 Hz, 1H), 2.53 (d, *J* = 4.9 Hz, 1H), 1.81–1.68 (m, 5H), 1.23 (s, 3H), 1.21–1.03 (m, 6H); ^13^C-NMR (CDCl_3_) δ 59.9, 53.6, 44.5, 28.9, 28.5, 26.4, 26.2, 26.1, 18.0; ESI-MS *m*/*z* 163 [M+Na]^+^; [α]_D_^26.0^ +9.1 (*c* 1.12, CHCl_3_). The enantiomeric excess of **4l** was determined after the similar conversion into 1,2,3,4-tetrahydroisoquinolinoalcohol by the similar procedure for **6b** synthesis. Chiral stationary-phase HPLC analysis: [DAICEL CHIRALPAK AD-H, *i*-PrOH/hexane 1/99, flow rate 1.0 mL/min, *t*_R_ 10.0 min (major, 97.8%) and 13.2 min (minor, 2.2%), detection at 220 nm].

#### 3.4.13. Ethyl 4-[(2*S*)-2-methyloxiran-2-yl]butanoate (**4m**)

Colorless oil; IR (neat) ν 2981, 1734, 1374, 1184, 1031, 798 cm^-1^; ^1^H-NMR (CDCl_3_) δ 4.12 (q, *J* = 7.3 Hz, 2H), 2.61 (d, *J* = 4.9 Hz, 1H), 2.57 (d, *J* = 4.9 Hz, 1H), 2.36–2.28 (m, 2H), 1.77–1.69 (m, 2H), 1.64–1.50 (m, 2H), 1.31 (s, 3H), 1.24 (t, *J* = 7.3 Hz, 3H); ^13^C-NMR (CDCl_3_) δ 173.3, 60.3, 56.5, 53.7, 35.9, 34.0, 20.8, 20.6, 14.2; ESI-MS *m*/*z* 195 [M+Na]^+^; HRMS [ESI] Calcd for C_9_H_16_NaO_3_^+^ [M+Na]^+^: 195.0997; Found 195.0991; The enantiomeric excess of **4m** was determined by chiral stationary-phase HPLC analysis [DAICEL CHIRALCEL OD, *i*-PrOH/hexane 0.5/99.5, flow rate 0.5 mL/min, *t*_R_ 21.2 min (minor, 4.6%) and 24.4 min (major, 95.4%), detection at 210 nm]; [α]_D_^26.8^ +6.6 (*c* 1.21, CHCl_3_).

#### 3.4.14. (2*S*)-2-Ethyl-2-phenyloxirane (**4n**) [3]

Colorless oil; IR (neat) ν 2971, 2937, 1448, 1083, 758, 700 cm^−1^; ^1^H-NMR (CDCl_3_) δ 7.38–7.32 (m, 4H), 7.29–7.27 (m, 1H), 2.98 (d, *J* = 5.5 Hz, 1H), 2.75 (d, *J* = 5.5 Hz, 1H), 2.20 (dq, *J* = 7.5, 14.5 Hz, 1H), 1.80 (dq, *J* = 7.5, 14.5 Hz, 1H), 0.94 (t, *J* = 7.5 Hz, 3H); ^13^C-NMR (CDCl_3_) δ 140.2, 128.5, 127.6, 126.2, 61.2, 55.5, 28.5, 9.2; ESI-MS *m*/*z* 171 [M+Na]^+^; The enantiomeric excess was determined by GC analysis [Chirasil DEX CB, injector temp. = 200 °C, detector temp. = 250 °C, column temp. = 70 °C (isothermic), t = 35.5 min (minor, 10.1%), 37.0 min (major, 89.9%)]; [α]_D_^27.1^ +25.8 (*c* 1.04, CHCl_3_). 

#### 3.4.15. (2*S*)-2-(4-Chlorophenyl)-2-ethyloxirane (**4o**)

Colorless oil; IR (neat) ν 2971, 2937, 1491, 1090, 911, 830 cm^−1^; ^1^H-NMR (CDCl_3_) δ 7.30 (s, 4H), 2.97 (d, *J* = 5.5 Hz, 1H), 2.70 (d, *J* = 5.5 Hz, 1H), 2.18 (dq, *J* = 14.9, 7.4 Hz, 1H), 1.78 (dq, *J* = 14.9, 7.4 Hz, 1H), 0.92 (t, *J* = 7.4 Hz, 3H); ^13^C-NMR (CDCl_3_) δ 138.6, 133.2, 128.4, 127.4, 60.5, 55.4, 28.1, 8.9; ESI-MS *m*/*z* 205 [M+Na]^+^; HRMS [ESI] Calcd for C_10_H_12_OCl^+ ^[M+H]^+^: 183.0571; Found 183.0568; The enantiomeric excess was determined by GC analysis [Chirasil DEX CB, injector temp. = 200 °C, detector temp. = 250 °C, column temp. = 110 °C (isothermic), t = 20.1 min (minor), 21.1 min (major)]; [α]_D_^21.7^ +11.2 (*c* 1.57, CHCl_3_).

#### 3.4.16. (2*S*)-2-(4-Bromophenyl)-2-ethyloxirane (**4p**)

Colorless oil; IR (neat) ν 2970, 2937, 1488, 1071, 910, 828 cm^−1^; ^1^H-NMR (CDCl_3_) δ 7.46 (d, *J* = 8.4 Hz, 2H), 7.24 (d, *J* = 8.4 Hz, 2H), 2.97 (d, *J* = 5.5 Hz, 1H), 2.69 (d, *J* = 5.5 Hz, 1H), 2.18 (dq, *J* = 14.9, 7.6 Hz, 1H), 1.76 (dq, *J* = 14.9, 7.6 Hz, 1H), 0.92 (t, *J* = 7.6 Hz, 3H); ^13^C-NMR (CDCl_3_) δ 139.1, 131.4, 127.8, 121.3, 60.5, 55.4, 28.0, 8.9; ESI-MS *m*/*z* 249 [M+Na]^+^; HRMS [ESI] Calcd for C_10_H_12_OBr^+ ^[M+H]^+^: 227.0066; Found 227.0067; The enantiomeric excess was determined by GC analysis [Chirasil DEX CB, injector temp. = 200 °C, detector temp. = 250 °C, column temp. = 130 °C (isothermic), t = 13.4 min (minor), 13.8 min (major)]; [α]_D_^23.1^ +20.6 (*c* 1.61, CHCl_3_).

#### 3.4.17. (2*S*)-2-(3-Chlorophenyl)-2-ethyloxirane (**4q**)

Colorless oil; IR (neat) ν 2971, 1598, 1463, 1080, 784, 695 cm^−1^; ^1^H-NMR (CDCl_3_) δ 7.37–7.36 (m, 1H), 7.28–7.26 (m, 3H), 2.99 (d, *J* = 5.2 Hz, 1H), 2.72 (d, *J* = 5.2 Hz, 1H), 2.21 (dq, *J* = 14.9, 7.6 Hz, 1H), 1.79 (dq, *J* = 14.9, 7.6 Hz, 1H), 0.95 (t, *J* = 7.6 Hz, 3H); ^13^C-NMR (CDCl_3_) δ 142.2, 134.3, 129.6, 127.6, 126.2, 124.2, 60.4, 55.4, 28.0, 8.9; ESI-MS *m*/*z* 205 [M+Na]^+^; HRMS [ESI] Calcd for C_10_H_12_OCl^+^ [M+H]^+^: 183.0571; Found 183.0571; The enantiomeric excess was determined by GC analysis [Chirasil DEX CB, injector temp. = 200 °C, detector temp. = 250 °C, column temp. = 100 °C (isothermic), t = 22.7 min (minor), 23.7 min (major) ]; [α]_D_^25.2^ +18.0 (*c* 1.55, CHCl_3_).

#### 3.4.18. (2*S*)-2-Ethyl-2-(2-fluorophenyl)oxirane (**4r**)

Colorless oil (88% yield, 67% *ee*); IR (neat) ν 2971, 2938, 1491, 1453, 1212, 757 cm^−1^; ^1^H-NMR (CDCl_3_) δ 7.39 (ddd, *J* = 15.0, 7.7, 1.9 Hz, 1H), 7.29–7.24 (m, 1H), 7.12 (ddd, *J* = 15.0, 7.4, 0.9 Hz, 1H), 7.04–7.00 (m, 1H), 2.99 (d, *J* = 5.2 Hz, 1H), 2.80 (d, *J* = 5.2 Hz, 1H), 2.10 (dq, *J* = 14.9, 7.7 Hz, 1H), 1.80 (dq, *J* = 14.9, 7.7 Hz, 1H), 0.90 (t, *J* = 7.7 Hz, 3H); ^13^C-NMR (CDCl_3_) δ 161.3+159.3, 129.3+129.2, 128.9+128.9, 127.5+127.4, 124.0+123.9, 115.2+115.1, 58.8, 53.4, 29.1, 8.9; ESI-MS *m*/*z* 189 [M+Na]^+^; HRMS [ESI] Calcd for C_10_H_12_OF^+ ^[M+H]^+^: 167.0867; Found 167.0866; The enantiomeric excess was determined by GC analysis [Chirasil DEX CB, injector temp. = 200 °C, detector temp. = 250 °C, column temp. = 80 °C (isothermic), t = 14.4 min (major), 15.4 min (minor)]; [α]_D_^25.6^ +45.3 (*c* 1.49, CHCl_3_).

#### 3.4.19. 3-[(2*S*)-2-Ethyloxiran-2-yl]pyridine (**4s**)

Colorless oil; IR (neat) ν 2971, 1418, 1023, 908, 808, 714 cm^−1^; ^1^H-NMR (CDCl_3_) δ 8.62 (d, *J* = 1.5 Hz, 1H), 8.51 (dd, *J* = 4.9, 1.5 Hz, 1H), 7.65 (ddd, *J* = 7.9, 2.2, 1.8 Hz, 1H), 7.25 (ddd, *J* = 7.9, 4.9, 0.7 Hz, 1H), 3.01 (d, *J* = 5.2 Hz, 1H), 2.74 (d, *J* = 5.2 Hz, 1H), 2.20 (dq, *J* = 14.9, 7.3 Hz, 1H), 1.82 (dq, *J* = 14.9, 7.3 Hz, 1H), 0.93 (t, *J* = 7.3 Hz, 3H); ^13^C-NMR (CDCl_3_) δ 148.7, 147.9, 135.6, 133.6, 123.1, 59.3, 55.1, 27.9, 8.8; ESI-MS *m*/*z* 172 [M+Na]^+^; HRMS [ESI] Calcd for C_9_H_12_ON^+ ^[M+H]^+^: 150.0913; Found 150.0910; The enantiomeric excess was determined by chiral stationary-phase HPLC analysis [DAICEL CHIRALCEL OD, *i*-PrOH/hexane 2/98, flow rate 0.5 mL/min, *t*_R_ 32.6 min (minor) and 54.0 min (major), detection at 254 nm]; [α]_D_^25.2^ +14.1 (*c* 1.65, CHCl_3_).

#### 3.4.20. (2*S*)-2-(4-Chlorophenyl)-2-propyloxirane (**4t**)

Colorless oil; IR (neat) ν 2960, 2934, 1491, 1092, 1013, 829 cm^−1^; ^1^H-NMR (CDCl_3_) δ 7.30 (s, 4H), 2.95 (d, *J* = 5.4 Hz, 1H), 2.68 (d, *J* = 5.4 Hz, 1H), 2.17–2.10 (m, 1H), 1.70–1.64 (m, 1H), 1.42–1.30 (m, 2H), 0.91 (t, *J* = 7.6 Hz, 3H); ^13^C-NMR (CDCl_3_) δ 138.8, 133.1, 128.4, 127.4, 59.9, 55.4, 37.4, 18.2, 14.1; ESI-MS *m*/*z* 219 [M+Na]^+^; HRMS [ESI] Calcd for C_11_H_14_OCl^+ ^[M+H]^+^: 197.0728; Found 197.0725; The enantiomeric excess was determined by GC analysis [Chirasil DEX CB, injector temp. = 200 °C, detector temp. = 250 °C, column temp. = 110 °C (isothermic), t = 28.9 min (minor), 29.6 min (major)]; [α]_D_^22.4^ +24.0 (*c* 1.55, CHCl_3_).

#### 3.4.21. (2*S*)-2-Phenyl-2-(propan-2-yl)oxirane (**4u**)

Colorless oil; IR (neat) ν 2965, 1468, 1383, 936, 760, 701 cm^−1^; ^1^H-NMR (CDCl_3_) δ 7.30–7.19 (m, 5H), 2.93 (d, *J* = 5.2 Hz, 1H), 2.65 (d, *J* = 5.2 Hz, 1H), 2.03 (qq, *J* = 7.1, 6.7 Hz, 1H), 0.88 (dd, *J* = 7.1, 6.7 Hz, 6H); ^13^C-NMR (CDCl_3_) δ 139.4, 127.9, 127.2, 64.5, 53.2, 33.1, 18.5, 17.8; ESI-MS *m*/*z* 185 [M+Na]^+^; HRMS [ESI] Calcd for C_11_H_15_O^+^ [M+H]^+^: 163.1117; Found 163.1116; The enantiomeric excess was determined by GC analysis [Chirasil DEX CB, injector temp. = 200 °C, detector temp. = 250 °C, column temp. = 80 °C (isothermic), t = 30.1 min (major), 31.1 min (minor)]; [α]_D_^23.1^ +25.8 (*c* 1.04, CHCl_3_).

### 3.5. Regioselective Ring-Oepning of Epoxide

#### 3.5.1. (2*S*)-1-Morpholin-4-yl-2-naphthalen-2-ylpropan-2-ol (**6b**)

A solution of epoxide **4b** (18,4 mg, 0.1 mmol) and morpholine (43.7 µL) in *i*PrOH (570 µL) was stirred at 90 °C for 72 h. After evaporating the solvent under reduced pressure, the residue was purified by flash column chromatography (SiO_2_, hexane/ethyl acetate = 5/1 to 1/1) to give **6b** (24.5 mg, 90% yield). No loss of enantioselectivity was confirmed by chiral stationary-phase HPLC analysis: colorless oil; IR (neat) ν 3421, 2965, 2851, 1455, 1116, 749 cm^−1^; ^1^H-NMR (CDCl_3_) δ 8.00 (brs, 1H), 7.85–7.79 (m, 3H), 7.50–7.43 (m, 3H), 4.47 (brs, 1H), 3.58–3.50 (m, 4H), 3.02 (d, *J* = 13.2 Hz, 1H), 2.67 (d, *J* = 13.2 Hz, 1H), 2.35 (brs, 2H), 2.29 (brs, 2H), 1.54 (s, 3H); ^13^C-NMR (CDCl_3_) δ 145.4, 133.2, 132.1, 128.0, 127.8, 127.4, 125.9, 125.6, 123.4, 123.1, 71.7, 69.3, 66.9, 54.9, 29.6; ESI-MS *m*/*z* 272 [M+H]^+^; HRMS [ESI] Calcd for C_17_H_22_O_2_N^+^ [M+H]^+^: 272.1651; Found 272.1649; The enantiomeric excess of **6b** was determined by chiral stationary-phase HPLC analysis [DAICEL CHIRALPAK AD-H, *i*-PrOH/hexane 1/9, flow rate 1.0 mL/min, *t*_R_ 8.3 min (minor) and 9.8 min (major), detection at 254 nm]; [α]_D_^26.2^ −13.8 (*c* 1.14, CHCl_3_).

#### 3.5.2. (2*R*)-2-Naphthalen-2-ylpent-4-yn-2-ol (**7b**)

To a solution of **4b** (18.4 mg, 0.1 mmol) and lithium acetylide ethylenediamine complex (102.3 mg, 0.1 mmol) in DMSO (1.0 mL) was stirred at room temperature for 1 h. The reaction mixture was cooled down at 0 °C and was quenched with H_2_O. The mixture was extracted with AcOEt (×2). The combined organic layers were washed with brine, and dried over Na_2_SO_4_. After evaporating solvent under reduced pressure, the residue was purified by flash column chromatography (SiO_2_, hexane/ethyl acetate = 10/1 to 3/1) to give **7b** (21.20 mg, >99% yield) as colorless oil. No loss of enantioselectivity was confirmed by chiral stationary-phase HPLC analysis; IR (neat) ν 3291, 3055, 2976, 1376, 1126, 748 cm^−1^; ^1^H-NMR (CDCl_3_) δ 7.97 (d, *J* = 1.9 Hz, 1H), 7.87–7.82 (m, 3H), 7.57 (dd, *J* = 1.9, 8.9 Hz, 1H), 7.50–7.47 (m, 2H), 2.89 (dd, *J* = 2.4, 16.5 Hz, 1H), 2.81 (dd, *J* = 2.8, 16.5 Hz, 1H), 2.51 (s, 1H), 2.05 (dd, *J* = 2.4, 2.8 Hz, 1H), 1.74 (s, 3H); ^13^C NMR (CDCl_3_) δ 143.6, 133.1, 132.5, 128.2, 128.0, 127.5, 126.1, 125.9, 123.3, 123.2, 8.3, 73.4, 71.9, 34.5, 29.2; ESI-MS *m*/*z* 233 [M+Na]^+^; HRMS [ESI] Calcd for C_15_H_14_ONa^+ ^[M+Na]^+^: 233.0942; Found 233.0936; The enantiomeric excess of **7b** was determined by chiral stationary-phase HPLC analysis [DAICEL CHIRALCEL OJ-H, *i*-PrOH/hexane 5/95, flow rate 1.0 mL/min, *t*_R_ 7.7 min (minor) and 11.6 min (major), detection at 254 nm]; [α]_D_^26.7^ +34.7 (*c* 1.18, CHCl_3_).

## 4. Conclusions

In summary, we have developed a catalytic asymmetric Corey-Chaykovsky epoxidation of ketones with dimethyloxosulfonium methylide (**2**) using an LLB **1a** + Ar_3_P=O complex. The reaction proceeded smoothly at room temperature and 2,2-disubstituted terminal epoxides were obtained in high enantioselectivity (97%–91% *ee*) and yield (>99%–88%) from a broad range of methyl ketones with 1–5 mol% catalyst loading. On the other hand, sterically more hindered ketones resulted in lower enantioselectivity. The enantioselectivity was strongly dependent on the steric hindrance of ketones, and aryl ethyl ketones gave products in moderate to good *ee* (up to 88% *ee*), while ketones with bulkier substituents resulted in less than 80% *ee*. The present method provides complementary approach to 2,2-disubstituted terminal epoxides in comparison with methods via epoxidation of alkenes.
